# Quality controls for antimicrobial disk diffusion testing on *Leptospira* Vanaporn Wuthiekanun agar

**DOI:** 10.1093/trstmh/trw076

**Published:** 2017-02-01

**Authors:** Vanaporn Wuthiekanun, Nicholas J. White, Premjit Amornchai, Nicholas P. J. Day, Direk Limmathurotsakul

**Affiliations:** a Mahidol-Oxford Tropical Medicine Research Unit, Faculty of Tropical Medicine, Mahidol University, Bangkok, 10400, Thailand; b Centre for Tropical Medicine and Global Health, Nuffield Department of Medicine, University of Oxford, Old Road Campus, Roosevelt Drive, Headington, Oxford, OX3 7FZ, UK; c Department of Tropical Hygiene, Faculty of Tropical Medicine, Mahidol University, Thailand

**Keywords:** Antimicrobial, Disk diffusion test, *Leptospira*, LVW Agar, Quality control strains.

## Abstract

**Background:**

Disk diffusion susceptibility testing for *Leptospira* spp. on *Leptospira* Vanaporn Wuthiekanun (LVW) solid agar was reported recently. However, it was unclear whether the zone sizes obtained on LVW agar were comparable with those of other bacteria on Mueller-Hinton agar.

**Methods:**

Here, we evaluate the disk diffusion assay on LVW agar using the standard quality control (QC) bacterial strains for 22 antimicrobials.

**Results:**

All antimicrobials provided zone sizes within the standard range for each QC bacterial strain, except for fosfomycin.

**Conclusions:**

In conclusion, the simple disk diffusion assay can be used to assess antimicrobial activity against *Leptospira* on LVW agar using standard bacterial strains for QC with the standard breakpoints (except for fosfomycin).

## Introduction

Leptospirosis is a worldwide zoonotic infection which is endemic in subtropical and tropical areas.^1^ Leptospiral infection in humans causes a wide range of symptoms. Most cases are mild, but severe and potentially fatal illness characterized by liver damage, kidney injury and bleeding may occur. Effective treatments include beta-lactam antibiotics and doxycycline. *Leptospira* spp. do not grow on conventional media, including Mueller-Hinton (MH) agar. Isolation of this organism from clinical specimens usually requires a specific medium (for example, Ellinghausen and McCullough modified Johnson and Harris [EMJH] liquid medium containing 3% rabbit serum, and semisolid EMJH medium containing 3% rabbit serum and 0.1% bacteriological agar) and considerable expertise, and it takes between 30 and 90 days. Antimicrobial susceptibility testing for *Leptospira* spp. has traditionally been performed by the broth macrodilution or microdilution technique.^2^ However, both methods are still labour-intensive and few antimicrobials had been tested against an array of *Leptospira* strains.^2^

We described recently a new solid agar, *Leptospira* Vanaporn Wuthiekanun (LVW) agar, which enables rapid growth and the isolation of single colonies of *Leptospira* spp.,^3^ and we reported the antimicrobial susceptibility testing of *Leptospira* spp. using disk diffusion on the LVW agar.^4^ Disk susceptibility testing is rapid and can evaluate with a wide range of antimicrobials. Using this method, we reported that *Leptospira* were susceptible to 17 antimicrobials (amoxicillin/clavulanic acid, amoxicillin, azithromycin, cefoxitin, ceftazidime, ceftriaxone, chloramphenicol, ciprofloxacin, clindamycin, doripenem, doxycycline, gentamicin, linezolid, nitrofurantoin, penicillin, piperacillin/tazobactam and tetracycline), were not susceptible to aztreonam, and were resistant to four antimicrobials (fosfomycin, nalidixic acid, rifampicin, and trimethoprim/sulfamethoxazole).^4^

However, it was unclear whether the zone sizes around the disks for other bacteria including those used for quality control (QC) purposes on LVW agar were comparable with those on MH agar. QC guidance for disk diffusion assays for *Leptospira* on LVW agar is needed. We have evaluated the inhibition zone sizes for standard QC bacterial strains on LVW agar. We used the Clinical and Laboratory Standard Institute (CLSI) performance standards for threshold zone sizes primarily for *Enterobacteriaceae*, and extended these to *Pseudomonas aeruginosa* or *Staphylococcus* spp. where zone sizes were not available for the drug being tested.^5^

## Materials and methods

We evaluated four QC bacterial strains on LVW agar. The antimicrobial agents selected for testing (n=22) represent the spectrum of drugs used in tropical settings for the treatment of suspected bacterial sepsis. The QC strains selected were *Escherichia coli* ATCC 25922, *E. coli* ATCC 35218 (recommended for evaluation of β-lactam/β-lactamase inhibitor combinations), *Pseudomonas aeruginosa* ATCC 27853 and *Staphylococcus aureus* ATCC 25923. All have well established susceptibility patterns using the CLSI methodology (M02-A12) and standard MH agar.^5^ All four QC bacterial strains were stored in trypticase soy broth (TSB) containing 15% glycerol at −80°C.

LVW agar was prepared as described previously^3^ and MH agar was prepared by pouring 25 ml into a 90 mm diameter petri dish to a depth of 4 mm.^5^ Since LVW agar was developed, it has been found useful to detect different morphologies for the isolation of *Leptospira* from the flood water,^6^ to maintain the *Leptospira* isolates without the need for frequent subculture,^7^ and to perform susceptibility testing using the disk diffusion method^4^ and Etest method.^3^

The QC isolates were recovered from freezer vials by streaking onto Columbia agar and incubating for 24 h at 37°C in air. Bacterial colonies were suspended in normal saline and adjusted to 0.5 McFarland standards to obtain approximate concentrations of 1–2×10^8^ CFU/ml. A sterile cotton swab was dipped into the adjusted suspension and rotated several times before pressed firmly on the inside wall of the tube to remove excess fluid. This was then inoculated the dried surface of MH and LVW agars for each QC strain using a rotator by moving the swab slowly from middle to the edge of the plate to ensure an even distribution of inoculum. Commercial antibiotic disks (Oxoid Ltd, Basingstoke, UK) were placed on the inoculated agar surface for maximum of six disks per plate for each QC isolate. Plates of MH agar and LVW agar with the same inoculation but without antibiotic disks were used as growth controls. All plates were incubated for 24 h at 30°C before determination of results. The zone diameters around each disk were interpreted using CLSI criteria. The test was performed in duplicate by two technicians at two independent time points.

For any antimicrobials which had zone sizes on LVW agar outside the standard range of the QC bacterial strain on MH agar, we reevaluated drug susceptibility of *Leptospira* on that antimicrobial using the broth microdilution susceptibility testing for the 10 pathogenic *Leptospira* isolates as described previously.^4^

## Results and Discussion

In general, the zone sizes of the standard QC bacterial strains on LVW agar were within their respective standard ranges on MH agar (Supplementary Tables 1–3).^5^ The only exception was fosfomycin which was larger. The zone size results from both technicians were consistent, with no more than 2 mm difference in zone size observed for any antibiotic.

The fosfomycin disk for *E. coli* ATCC 25922 on LVW agar gave a larger inhibition zone (at 47 mm) than the standard range on MH agar (22–30 mm), while the fosfomycin zone for *S. aureus* ATCC 25923 on LVW agar (at 25 mm) was within its standard range on MH agar (25–33 mm). We reported previously that all 10 pathogenic *Leptospira* spp. isolates had no zone of inhibition around the fosfomycin disk.^4^ We additionally performed a broth microdilution assay and found that the minimum inhibitory concentration (MIC) of all 10 *Leptospira* isolates for fosfomycin was ≥256 µg/ml. This confirms that *Leptospira* are resistant to fosfomycin.

Routine internal QC testing with a range of QC strains is a major component of the quality assurance process, to monitor performance of the test.^5^ Disk susceptibility testing of *Leptospira* spp. on LVW agar is a new approach and so there is a need to ensure that other bacteria including those used for QC proposes similar zone sizes on LVW agar and MH agar. We have demonstrated that, using LVW agar, all four CLSI QC bacterial strains gave zone sizes within their respective standard ranges on MH agar for all antimicrobials except for fosfomycin. Therefore the disk diffusion assay on LVW agar should not be used for determining fosfomycin susceptibility of *Leptospira* strains (although all strains tested to date have been fosfomycin resistant). A limitation of this study is that our study used an incubation temperature of 30°C rather than 35°C, which was recommended by CLSI, because 30°C was used for the disk susceptibility testing of *Leptospira* spp. on LVW agar.^4^ We used a standard incubation period for the QC bacteria on MH agar (24 h) rather than two plus five days, which was for *Leptospira* spp., because all QC strains grow comparably on both LVW and MH agar, while *Leptospira* spp. is still a comparatively slow-growing bacterium even on LVW agar.

### Conclusions

We conclude that these four QC strains can be used for quality assessment using the method recommended while tests are being performed for *Leptospira* spp. New antimicrobials to be tested against *Leptospira* spp. on LVW agar will need validation against QC bacterial strains to confirm adequate diffusion through the LVW agar.

## Supplementary data

Supplementary data are available at *Transactions of The Royal Society of Tropical Medicine and Hygiene* online.

## Supplementary Material

Supplementary Data
