# Supplementary material for: Quality controls for antimicrobial disk diffusion testing on Leptospira Vanaporn Wuthiekanun agar
Source: Trans R Soc Trop Med Hyg. 2017 Feb 1;110(11):673–5. doi: 10.1093/trstmh/trw076 (PMC5412067; doi:10.1093/trstmh/trw076)
Supplement: Supplementary Data [file ED_Supp_tables.docx]

**Supplementary Table 1.** Zone diameter (millimeters) of *Escherichia coli* ATCC 25922 and *Escherichia coli* ATCC 35218 on Muller Hinton (MH) agar and *Leptospira* Vanaporn Wuthiekanun (LVW) agar

|  |  | *E. coli ATCC 25922* | *E. coli ATCC 25922* | *E. coli ATCC 25922* |
| --- | --- | --- | --- | --- |
| Antimicrobials | Disk content (µg) | Control range | MH agar (mean)^a^ | LVW agar (mean)^a^ |
| Amoxicillin/clavulanic acid | 20/10 | 18–24 | 21 | 24 |
| Amoxicillin/clavulanic acid^b^ | 20/10 | 17–22 | 20 | 22 |
| Amoxicillin | 10 | 15–22 | 16 | 22 |
| Aztreonam | 30 | 28–36 | 30 | 30 |
| Cefoxitin | 30 | 23–29 | 24 | 29 |
| Ceftazidime | 30 | 25–32 | 29 | 32 |
| Ceftriaxone | 30 | 29–35 | 30 | 29 |
| Chloramphenicol | 30 | 21–27 | 24 | 23 |
| Ciprofloxacin | 5 | 30–40 | 31 | 32 |
| Trimmethoprim/sulfamethoxazole | 1.25/23.75 | 23–29 | 29 | 27 |
| Doripenem | 10 | 27–35 | 31 | 31 |
| Doxycycline | 30 | 18–24 | 22 | 19 |
| Fosfomycin | 50 | 22–30 | 30 | 47^c^ |
| Gentamicin | 10 | 19–26 | 19 | 23 |
| Nalidixic acid | 30 | 22–28 | 27 | 23 |
| Nitrofurantoin | 300 | 20–25 | 23 | 22 |
| Piperacillin/tazobactam | 100/10 | 24–30 | 27 | 26 |
| Piperacillin/tazobactam^b^ | 100/10 | 24–30 | 26 | 26 |
| Rifampicin | 5 | 8–10 | 9 | 10 |
| Tetracycline | 30 | 18–25 | 24 | 25 |

^a^ Mean of four values (round up if ≥0.5).

^b^ Tested with *Escherichia coli* ATCC 35218 recommended for β-lactam/β-lactamase inhibitor combinations.

^c^ *Escherichia coli* ATCC 25922 on LVW agar gave an inhibition zone at 47mm, larger than the standard range (22–30 mm).

**Supplementary Table 2.** Zone diameter (millimeters) of the *Pseudomonas aeruginosa* ATCC 27853 on Muller Hinton (MH) agar and *Leptospira* Vanaporn Wuthiekanun (LVW)agar

|  |  | *P.aeruginosa ATCC 27853* | *P.aeruginosa ATCC 27853* | *P.aeruginosa ATCC 27853* |
| --- | --- | --- | --- | --- |
| Antimicrobials | Concentration | Control range | MH agar (mean)^a^ | LVW agar (mean)^a^ |
| Aztreonam | 30µg | 23–29 | 25 | 27 |
| Ceftazidime | 30µg | 22–29 | 28 | 29 |
| Ceftriaxone | 30µg | 17–23 | 21 | 20 |
| Ciprofloxacin | 5µg | 25–33 | 27 | 27 |
| Doripenem | 10µg | 28–35 | 33 | 28 |
| Gentamicin | 10µg | 17–23 | 20 | 23 |
| Piperacillin/tazobactam | 100/10µg | 25–33 | 29 | 31 |

^a^Mean of four values (round up if ≥0.5).

**Supplementary Table 3.** Zone diameter (millimeters) of the *Staphylococcus aureus* ATCC 25923 on Muller Hinton agar (MH) and *Leptospira* Vanaporn Wuthiekanun agar (LVW)

|  |  | *S. aureus ATCC 25923* | *S. aureus ATCC 25923* | *S. aureus ATCC 25923* |
| --- | --- | --- | --- | --- |
| Antimicrobials | Concentration | Control range | MH agar (mean)^a^ | LVW agar (mean)^a^ |
| Amoxicillin/clavulanic acid | 20/10 µg | 28–36 | 33 | 29 |
| Amoxicillin | 10µg | 27–35 | 30 | 27 |
| Azithromycin | 15µg | 21–26 | 22 | 23 |
| Cefoxitin | 30µg | 23–29 | 27 | 23 |
| Ceftazidime | 30µg | 16–20 | 20 | 17 |
| Ceftriaxone | 30µg | 22–28 | 26 | 22 |
| Chloramphenicol | 30µg | 19–26 | 21 | 19 |
| Ciprofloxacin | 5µg | 22–30 | 27 | 28 |
| Clindamycin | 2µg | 24–30 | 25 | 24 |
| Trimmethoprim/sulfamethoxazole | 1.25/23.75µg | 24–32 | 30 | 24 |
| Doripenem | 10µg | 33–42 | 35 | 35 |
| Doxycycline | 30µg | 23–29 | 27 | 24 |
| Fosfomycin | 50µg | 25–33 | 26 | 26 |
| Gentamicin | 10µg | 19–27 | 24 | 24 |
| Linezolid | 30µg | 25–32 | 24 | 24 |
| Nitrofurantoin | 300µg | 18–22 | 21 | 20 |
| Penicillin | 10 Units | 26–37 | 33 | 28 |
| Piperacillin/tazobactam | 100/10µg | 27–36 | 31 | 28 |
| Rifampicin | 5µg | 26–34 | 32 | 26 |
| Tetracycline | 30µg | 24–30 | 26 | 23 |

^a^Mean of four values (round up if ≥0.5).
